# Development and Structural Modification of BACE1 Inhibitors

**DOI:** 10.3390/molecules22010004

**Published:** 2016-12-22

**Authors:** Ting Gu, Wen-Yu Wu, Ze-Xi Dong, Shao-Peng Yu, Ying Sun, Yue Zhong, Yu-Ting Lu, Nian-Guang Li

**Affiliations:** Industrialization and Formulae Innovative Medicine, Nanjing University of Chinese Medicine, Nanjing 210023, Jiangsu, China; guting1992@163.com (T.G.); 15150513147@163.com (W.-Y.W.); dongzexi1215@163.com (Z.-X.D.); yushaopeng0405@163.com (S.-P.Y.); 18260028601@163.com (Y.S.); 18260028701@163.com (Y.Z.); lyt1996tian@outlook.com (Y.-T.L.)

**Keywords:** Alzheimer’s disease, β-amyloid, BACE1, BACE1 inhibitors, structural modification

## Abstract

Alzheimer’s disease (AD) is a progressive neurodegenerative disorder which usually occurs in the elderly. The accumulation of β-amyloid and the formation of neurofibrillary tangles are considered as the main pathogenies of AD. Research suggests that β-secretase 1 (BACE1) plays an important role in the formation of β-amyloid. Discovery of new BACE1 inhibitors has become a significant method to slow down the progression of AD or even cure this kind of disease. This review summarizes the different types and the structural modification of these new BACE1 inhibitors.

## 1. Introduction

Alzheimer’s disease (AD), which is a frequent and progressive form of neurodegenerative disorder, is relatively common in the elderly. AD affects 13% in the population over the age of 65, and this proportion rises to 45% over the age of 85 [1]. AD chronically ravages the cognitive capabilities of patients and leads to a decline in the ability of cognition and memory [2]. According to the statistics, worldwide there were about 47 million documented cases in 2015, and this will reach 107 million in 2050 if no new medicine to delay the disease progression becomes available [3]. Due to the rapid growing number of cases and the lack of available effective therapies, AD represents a serious social, economic, and political burden in many countries. Thus, the development and application of new drugs to cure AD or even stop the disease progression have attracted more attention globally.

Preclinical AD, mild cognitive impairment (MCI) and dementia are the three main stages during the pathological progress of AD [4]. In clinical practice, neuroimaging, enzyme-linked immunosorbent assay (ELISA), and polymerase chain reaction (PCR) are applied to detect Aβ to diagnose AD. However, these methods are not applied widely [5] because their diagnostic equipment and operational requirements are very tough. The diagnosis of preclinical AD, which is a spectrum ranging from the patients at risk of developing AD to those with subtle cognitive symptoms, but not severe enough to warrant an MCI diagnosis [4], requires a suitable biomarker.

Besides the difficulties in diagnosis, there are no approved therapies for AD to minimize disease progression [6]. Although cholinesterase inhibitors such as donepezil, rivastigmine, galantamine, and memantine are drugs commonly used to prevent glutamate-mediated neurotoxicity on the market, they can only slow down the disease progression in the short term, and fundamentally they cannot cure AD [7]. Thus, finding an effective way to completely cure AD is an imminent problem.

Typical pathologic features of AD include: (1) the accumulation of β-amyloid (Aβ); (2) the formation of neurofibrillary tangles (NFTs); (3) the degeneration of vascular and the loss of neurons; (4) hyperphosphorylated tau protein [8,9,10,11]. Among these pathological changes, Aβ40 and Aβ42 play important roles in the pathogenesis of AD. With these two peptides, various kinds of oligomeric structures including protofibrils, fibrils, and plaques can be formed [4]. According to the literature, Aβ oligomers create a hazard to the neurons, particularly to the neuronal synapses [2].

Since the significance of Aβ is recognized, the biosynthesis pathway has also been investigated. β-Secretase 1 (BACE1) and γ-secretase are two proteases which cleave amyloid precursor protein (APP) into Aβ. BACE1 initially cleaves APP at the N-terminus of the Aβ peptide domain, followed by the cleavage in the transmembrane domain of APP by γ-secretase, thus leading to the secretion of Aβ peptide [12]. BACE1, as the initial enzyme of the formation of Aβ, exists widely in the brain of AD patients [13]. Furthermore, Aβ cannot be detected in neurons in BACE1 knockout rats [14], so BACE1 has been regarded as a novel target for AD therapy.

In this review, BACE1 inhibitors with peptidomimetic, piperazine and amino/imino structures are summarized. In addition, some natural products with BACE1 inhibitory activities are also introduced. This study should provide important information for the design and structural modification of BACE1 inhibitors.

## 2. BACE1 Inhibitors with Peptidomimetic Structure

The research on BACE1 inhibitors had been lasted for decades of years. In 1999, Sinha confirmed that BACE1 is a membrane-bound aspartic proteinase with 501 amino acids according to biology assays [15]. It also plays an important role in the formation of Aβ as a rate-limiting enzyme [16].

**Figure 1:**
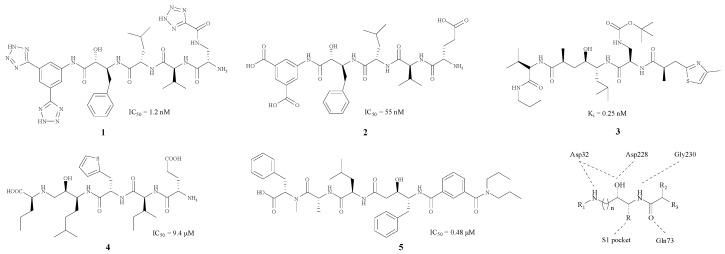
BACE1 inhibitors with peptidomimetic structure.

Originally, BACE1 inhibitors were projected as polypeptides due to the aspartic acid, which is the substrate of the enzyme (**1**–**5**) [17,18,19,20,21]. The structures and the corresponding IC_50_ values are revealed in Figure 1.

As Figure 1 shows, each of these compounds contains an imino group and a hydroxy in the centre of the structure. Molecular docking studies indicate that these two groups are capable of combining with Asp228 and Asp32, which are two important amino acid residues in BACE1. Large groups such as benzyl or isopentyl groupd, usually appear near the hydroxyl, and could combine with the S1 pocket. Thr72, Thr232 and Gln73 are hydrogen bond donors (HBD) in BACE1, whereas the carbonyl group, as a hydrogen bond acceptor (HBA), has a good connection to Thr232 and Gln73 residues. Furthermore, Gly230 could combine with another imino moiety as a HBA.

In BACE1 inhibitory assays, the five compounds **1**–**5** [17,18,19,20,21] shown in Figure 1 all exhibited good inhibitory effects, with IC_50_ values ranging from 0.48 to 55 nM. Nevertheless, this kind of BACE1 inhibitors did not perform well in clinical experiments. Because of the presence of numerous HBD and HBA, these inhibitors with high total polar surface area (TPSA) could not pass through the blood brain barrier (BBB) successfully [22], so the improvement of these structures so they can pass through the BBB should be carried out, and some hydrophobic groups such as a phenyl group can be introduced in order to reduce the polarity of these compounds.

**Figure 2:**
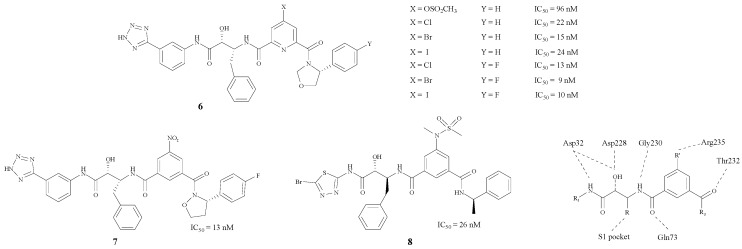
Structures of peptidomimetic BACE1 inhibitors with phenyl groups.

Hamada et al. have concentrated on BACE1 inhibitors for several years. They modified the structure by inserting a phenyl group in between the two carbonyl groups. Moreover, the protein model showed that Arg235 played an important role in the pocket according to the X-ray crystal structures [23], as the σ–π interaction between the inhibitor and the BACE1-Arg235 side chain exerts an enormous effect on the inhibition mechanism [24]. By changing the R’ group, a series of compounds **6**–**8** (Figure 2) with hydroxymethylcarbonyl (HMC) isosteres was synthesized [24,25,26,27,28]. The results showed that compound **6** (X = Br) could bond best with the BACE1-Arg235 side chain. In addition, the introduction of a fluorine atom in the side chain on the phenyl could improve BACE1 inhibitory activities. There is also a tetrazole on the side chain of the structure (**6**, Y = F) which may improve the penetrability across the BBB. Different structural modifications on the tetrazole could lead to disparate IC_50_ values.

**Figure 3:**
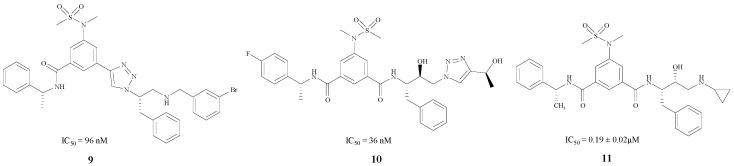
The BACE1 inhibitors with peptidomimetic structures.

Monceaux, Zou and Simone synthesized BACE1 inhibitors **9**, **10** and **11** [29,30,31] (Figure 3), with IC_50_ values ranging from 36 to 192 nM. Especially compound **9** [29], which has a triazole-substituted amido bond with Gly230 and Gln73, showed good inhibitory activity with an IC_50_ of 96 nM.

However, as the active site of β-secretase is less hydrophobic than other human aspartic proteases [32], only compounds with low polarity could penetrate the BBB [33]. BACE1 inhibitors with peptidomimetic structures obviously fail to meet this requirement because of their excessive number of HBAs and HBDs. Therefore, the design of novel BACE1 inhibitors is very important.

## 3. BACE1 Inhibitors with Piperazine Structure

**Figure 4:**
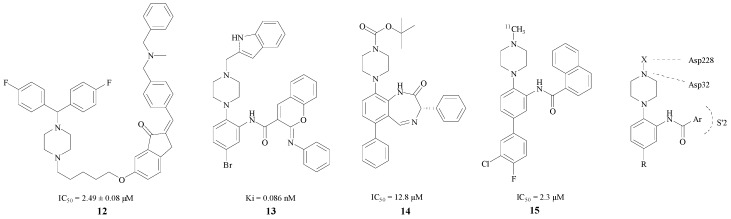
BACE1 inhibitors with piperazine rings.

Based on the fact that the H bond in peptidomimetic inhibitors could interact with the Gly34, Gly230, Thr230 or Thr232 [34], Rampa et al. introduced a piperazine ring into BACE1 inhibitors. Compound **12** was found to have remarkable effect and an IC_50_ = 2.49 ± 0.08 μM [35] (Figure 4). The attachment of the phenyl ring could lead to a valid hydrophobic interaction, which would increase the probability of permeability into the brain. Thus, many BACE1 inhibitors were inspired and designed as piperazine-based biphenyl analogs.

Edraki et al. considered a type of biaryl naphthalenes with piperzines as a lead compound. Through the methods of molecular docking and structural modification, Edraki et al. obtained compound **13** with a Ki value of 0.086 nM. In addition, according to a computational study, the π–π stacking interaction between the phenyl-imino group and Phe108 added stability with the enzyme [34]. Butini and Kawai also obtained two series of BACE1 inhibitors, respectively, and the best compounds **14**, **15** of each series, shown in Figure 4, exhibited IC_50_ values of 12.8 and 2.3 μM [36,37]. The interaction between BACE1 and the piperazine ring is also shown in Figure 4 [34]. One of the nitrogen in the piperazine combines with Asp32 through a hydrogen bond, and the substituent on this nitrogen connected with Asp228 as well. Furthermore, the Ar group bonds with the S′2 pocket preferably. Nevertheless, this structure is not suitable for all inhibitors. A more compact basic structure should be confirmed in BACE1 inhibitor design.

## 4. BACE1 Inhibitors with Amino/Imino Structures

Novel methods such as substrate-based design, high-throughput screening (HTS) and fragment-based approaches have been applied in drug discovery [32]. Moreover, as BACE1 X-ray crystal structures exhibited high flexibility [38], the structures of BACE1 inhibitors could be variously modified. In the molecular docking of the BACE1 inhibitors with polypeptides, the aspartic acids Asp228 and Asp32 acted as two important HBDs, and combined with the amino or the imino through hydrogen bonds. Based on this docking study, several kinds of small molecule inhibitors could be designed.

**Figure 5:**
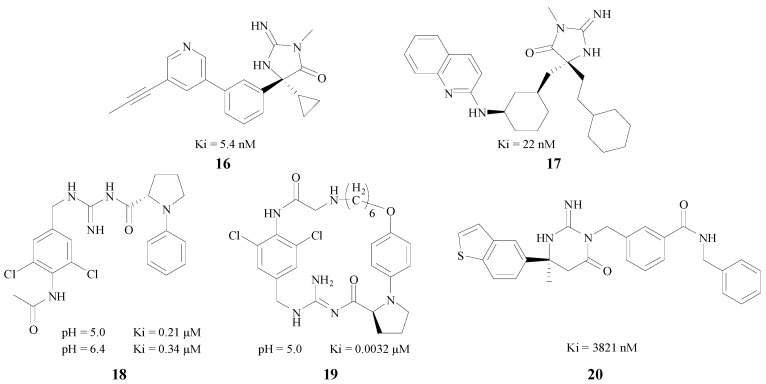
BACE1 inhibitors with amino/imino structures.

Cumming et al. projected a series of cyclic acylguanidine BACE1 inhibitors based on the fact that the diphenyliminohydantoin group had the features of high ligand efficiency (LE), inherent selectivity over the related aspartyl protease cathepsin D, and favorable pharmacokinetic properties [39]. Caldwell and Boy both obtained series of BACE1 inhibitors by the means of fragment screening. The former used the iminohydantoin and the latter identified an acyl guanidine (AG) as the cores, respectively [40,41]. The Ki values of the compounds with best inhibition, **16** and **17** (Figure 5) were 5.4 nM and 22 nM, respectively. Boy tested two types of inhibitors under different pH values, and the Ki of 18, the best acyclic one (Figure 5) was 0.21 μM at pH 5.0, and 0.34 μM at pH 6.4. In addition, the macrocyclic acyl guanidine inhibitor **19** (Figure 5) gave a Ki value of 0.0032 μM at pH 5.0. Mckittrick et al. designed a BACE1 inhibitor **20** with a guanidine portion as the kernel in an iminopyrimidinone skeleton (Figure 5), which could bind with the catalytic aspartic acids in the active site of the BACE1 [42]. This compound also had a good inhibitory activity (Ki = 3821 nM).

Since a large variety of BACE1 inhibitors emulated the same active site interactions, the enzyme pockets like S1, S3, and S2′ also play an important role in inhibition besides the combination of amino and aspartate residues [43]. Several researchers have focused on this type of compounds with a pentatomic or hexatomic ring. In addition, the inhibitors could match the enzyme pockets better by introducing substituted phenyls and other aromatic groups.

**Figure 6:**
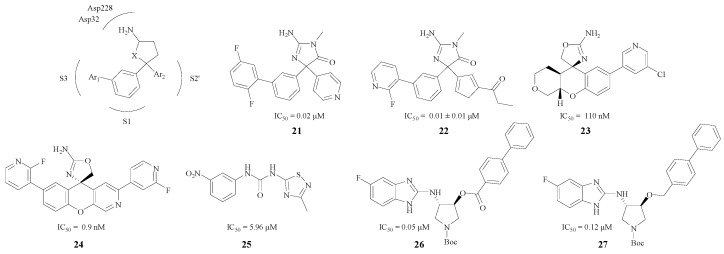
Pentatomic ring BACE1 inhibitors with amino structures.

Characteristic pentatomic rings in BACE1 inhibitors are presented in Figure 6. The amino and the heteroatom, act as an HBA or an HBD and bind with Asp228 and Asp32 very efficiently. Firstly, the phenyl ring was an excellent linker which could both keep the compound in the S1 pocket and lead the Ar_1_ to the S3 pocket. Secondly, Ar_2_ could combine with the S2′ pocket efficiently, so this kind of structure may possess a remarkable BACE1 inhibitory activity. For instance, the interaction between the pyridine nitrogen and Trp76 in compound **21** (Figure 6) was a key feature in the S2′ region of the enzyme that contributed to the increased potency [44]. Malamas et al. designed **22** (Figure 6), a type of BACE1 inhibitor with a pyrazolyl ring and a pyrimidine as well, which enhanced the selectivity against BACE2 and cathepsin D [45]. Moreover, Volgraf and Chen made the phenyl ring and the Ar_2_ connect with an oxygen atom respectively, and this could also increase the inhibitory effect (compound **23** with an IC_50_ value of 110 nM and compound **24** with an IC_50_ value 0.9 nM) [43,46]. Besides, different structure modifications were made in pentatomic rings (compounds **25**, **26**, **27**, Figure 6), and these compounds all showed good BACE1 inhibitory activities as their IC_50_ values ranged from 0.05 μM to 5.96 μM [47,48].

**Figure 7:**
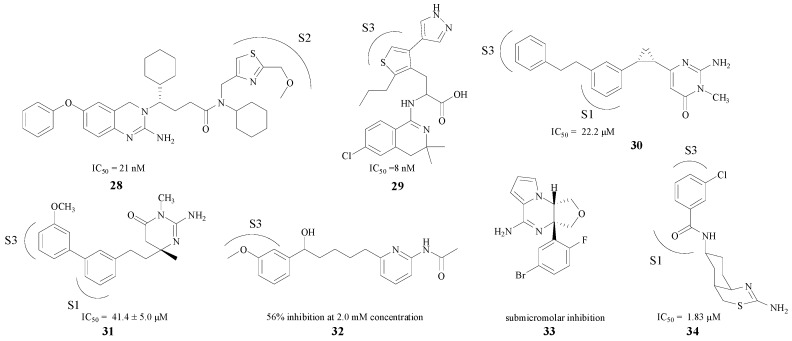
Hexatomic rings BACE1 inhibitors with amino structures.

Compared with the pentatomic rings, the modifications in hexatomic rings put more emphasis on the combination between compounds with S1/S3 pockets (e.g., **28**–**34**). Seven BACE1 inhibitors are presented in Figure 7 [49,50,51,52,53,54,55]. Most of these compounds displayed potent in vitro activities against BACE1, with IC_50_ values ranging from low micromolar to sub-micromolar concentrations (8 nM–46.4 μM). Compound **32** exhibited 56% rBACE1 inhibitory activity at 2.0 mM concentration as well [53]. Based on the fact that a hydrogen bond in the amino heterocycle ring could interact with Asp228 and Asp32, most of these structures improved their inhibitory activities by stretching the structure into the S3 pocket. The introduction of a core aromatic group could bind at the entrance to the hydrophobic S3 pocket. Furthermore, chloro and other substituent on the aromatic ring could extend into the pocket [55], and may bind with the target firmly.

## 5. BACE1 Inhibitors from Natural Products

Besides synthesized compounds, the study of metabolites from flora and fauna is a research hotspot in searching for novel lead compounds for AD treatment. During the research on AD, several kinds of natural products have been found. Curcumin and its derivatives, flavonoids, terpenes, ginsenosides, alkaloids and other natural compounds are widely used for the treatment of AD, and some of them have shown promising results.

**Figure 8:**
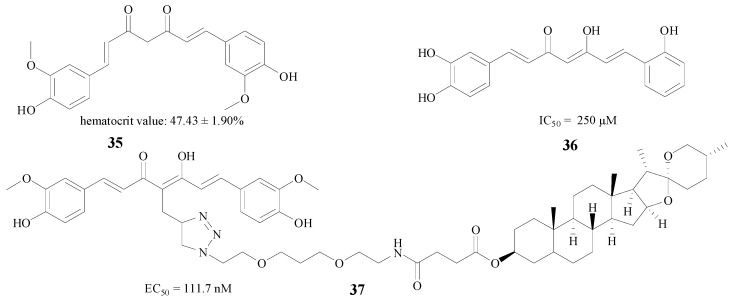
Structures of curcumin and its derivatives.

Curcumin, which derives from the spice turmeric, is extensively applied in AD therapy [56]. Samy et al. used three methods, including erythropoietin individually, curcumin (**35**, Figure 8) individually, and the two compounds combined in a neurobehavioral test, and the results showed that both compounds and their mixture efficiently reduced hippocampal β-amyloid accumulation [57]. Besides the combination therapy, structural modification is also a good method to improve efficacy. Konno et al. substituted a hydrogen atom and a hydroxy moiety by methoxy groups, and the new compound **36** (Figure 8) showed an IC_50_ value of 250 μM against rBACE1 enzyme [58]. Interestingly, Chojnacki et al. connected curcumin with diosgenin to afford new compound **37**; this structure proved very potent, with an EC_50_ of 111.7 nM in MC65 neuroblastoma cells [59] (Figure 8).

**Figure 9:**
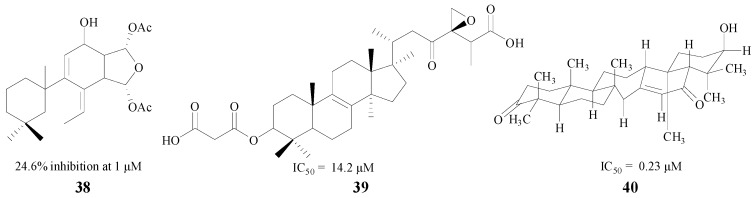
Structures of terpenoids.

Terpenoids are another type of natural product with the ability to inhibit BACE1. Diterpenes and triterpenes are two common structures used in medical treatment. Leirós et al. found four diterpenes from gracilins, which were *Spongionella*-derived compounds. One of them (**38**, Figure 9) could produce a potent inhibition against BACE1, and decrease BACE1 activity by 24.6% at 1 μM [60]. Triterpenes also have significant therapeutic effects on AD, and Sorribas and Nguyen extracted and separated triterpenes **39** and **40**. Individually, both of them showed good inhibitory activity with IC_50_ values of 14.2 μM and 0.23 μM, respectively [61,62] (Figure 9).

**Figure 10:**
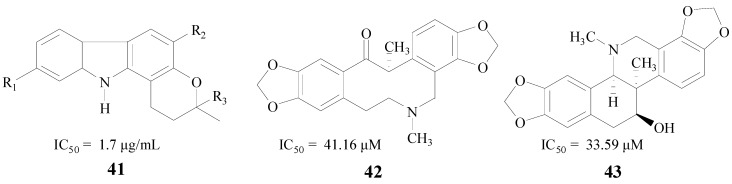
Structures of alkaloids.

Alkaloids, which occur widely in organisms, especially in plants, are very interesting for their diverse biological activities. Mani et al. found that the total alkaloidal extract from *Murraya koenigii* leaves (MKA) (**41**, Figure 10) was beneficial to cognitive functions. The IC_50_ value of MKA against BACE1 was 1.7 μg/mL [63]. Chlebek et al. also obtained seven alkaloids which were active in inhibiting BACE1, (−)-corycavamine (**42**) and (+)-corynoline (**43**) were two compounds with IC_50_ values of 41.16 μM and 33.59 μM, respectively [64] (Figure 10).

**Figure 11:**
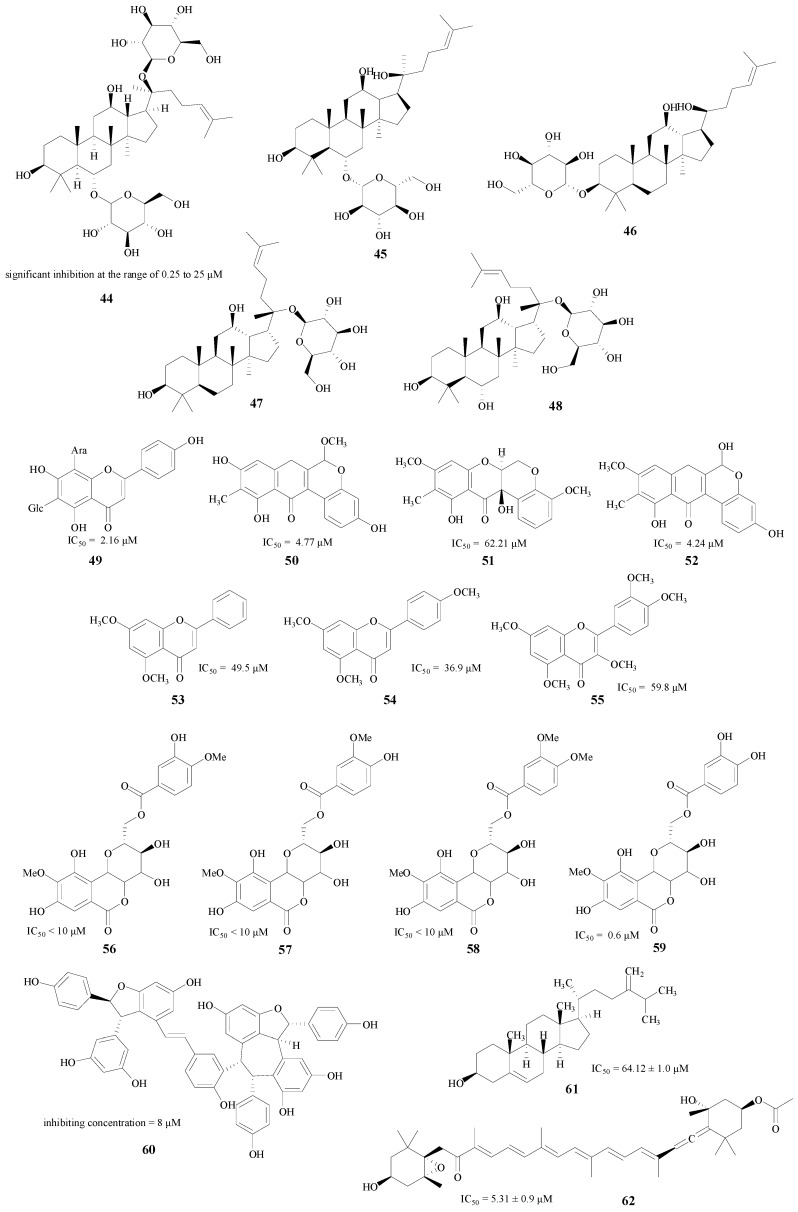
Structures of other natural products.

In addition, there are also a lot of other natural product classes which have inhibitory abilities against BACE1 enzyme, such as the ginsenosides [65,66], flavonoids [67,68,69], bergenin [70], polyphenols [71] and sterols [72] (compounds **44**–**62**, Figure 11). These compounds may be lead compounds for the synthesis and structural modification of novel BACE1 inhibitors.

## 6. Conclusions

As a progressive neurodegenerative disorder, AD has raised abundant concern from the public due to its burden on family and society. According to the research of the mechanism of AD, BACE1 is considered as a key enzyme that participates in the formation of Aβ, which broadly exists in the brains of patients with AD. Compounds with peptidomimetic structures are effective in BACE1 inhibition according to aspartic proteinase results in biology experiments in vitro. Nevertheless, this kind of BACE1 inhibitors didn’t perform well in pre-clinical trials due to their excessive number of hydrogen bond donors and acceptors, which increase the polarity and further lead to a lack of permeability across the BBB.

Based on molecular docking studies using the amino acid residues in BACE1 enzyme Asp228 and Asp32 play an important role in the interactions between compounds and the enzyme. Furthermore, S1, S3, S2′ and other pockets also exhibited a central role in binding with the BACE1 inhibitors. In the light of these studies, compounds with amino heterocycles were designed and synthesized. The presence of amino and aromatic rings maintained the inhibitory ability and decreased the polarity of the structure at the same time.

Natural products are an important resource which could provide lead compounds for BACE1 inhibitor discovery, and novel lead compounds could be obtained by extracting and separating them from plants. Taken together, all this evidence indicates that the development and structural modification of BACE1 inhibitors could have a great influence in the treatment of AD.
